# Demographics and Outcomes Related to Wilson’s Disease Patients: A Nationwide Inpatient Cohort Study

**DOI:** 10.7759/cureus.44714

**Published:** 2023-09-05

**Authors:** Ankoor H Patel, Meghana Ghattu, Natale Mazzaferro, Alexander Chen, Kaitlyn Catalano, Carlos D Minacapelli, Vinod Rustgi

**Affiliations:** 1 Internal Medicine, Rutgers Robert Wood Johnson Medical School, New Brunswick, USA; 2 Medicine, Division of Gastroenterology and Hepatology, Rutgers Robert Wood Johnson Medical school, New Brunswick, USA; 3 Biostatistics and Epidemiology, Rutgers Robert Wood Johnson Medical School, New Brunswick, USA; 4 Medicine, Rutgers Robert Wood Johnson Medical School, New Brunswick, USA; 5 Medicine, Division of Gastroenterology and Hepatology, Rutgers Robert Wood Johnson Medical School, New Brunswick, USA

**Keywords:** copper, liver transplant, liver disease, national inpatient sample, wilson’s disease

## Abstract

Background and objective

Wilson’s disease (WD) is a rare autosomal recessive disease caused by mutations in the ATP7B gene, leading to impairment in copper excretion and subsequent accumulation primarily in the liver and brain. There is scarce data in the literature on the outcomes and cost burden of WD. In light of this, we aimed to assess outcomes, mortality rates, and costs associated with WD patients and their management in the United States (US).

Methods

We conducted a retrospective cohort study based on data in the National Inpatient Sample (NIS) database from 2007 to 2017. A total of 17,713 patients with a diagnosis of WD were identified using the International Classification of Diseases, Ninth or Tenth Revision (ICD-9/10) codes. Bivariate analyses were performed using t-tests for continuous variables and Pearson’s chi-square tests for categorical variables, where two-sided p-values <0.05 were considered statistically significant.

Results

The majority of the 17,713 identified patients were female. The mean age of the WD cohort was 49 years. WD patients had a higher prevalence of Kayser-Fleischer rings, neuropsychiatric symptoms, and liver-related complications including acute hepatitis, liver failure, portal hypertension, and cirrhosis. Peptic ulcer disease, connective tissue disease, and hemolytic anemia were significantly more common in the WD cohort. Compared to the non-WD cohort, the WD cohort had a significantly higher mortality rate, longer length of stay (LOS), and increased hospitalization costs (p<0.0001). A higher proportion of patients who had undergone orthotopic liver transplantation (OLTx) were in the 18-34 and 35-44-year-old subgroups. On the contrary, the highest proportion of patients with WD who had not undergone OLTx were in the 55-89-year-old subgroup. WD patients who had undergone OLTx had a lower degree of comorbidities, decreased mortality rate, and shorter LOS (all p<0.0001) compared to WD patients who had not undergone OLTx.

Conclusion

Based on our findings, patients with WD had a higher LOS, mean hospitalization costs, and mortality rate compared to the non-WD cohort. Mortality rate and LOS were significantly lower in WD patients who had undergone OLTx.

## Introduction

Wilson's disease (WD) is a rare autosomal recessive condition affecting up to one in every 30,000 individuals in the United States (US) [1]. WD is caused by mutations in the ATP7B gene that encodes a copper-transporting ATPase [2]. Deficiency of this protein leads to impaired copper excretion and subsequent copper accumulation primarily in the liver and the brain. The main clinical manifestations of WD are due to complications of hepatic involvement, including cirrhosis, and neuropsychiatric disturbances [3]. Untreated WD can have fatal outcomes [4].

According to current clinical practice guidelines, the mainstay of treatment for WD involves limiting intestinal absorption of copper using zinc or promoting its extraction with chelators such as D-penicillamine and trientine [3,5,6]. WD patients with acute liver failure or decompensated cirrhosis that does not respond to medical management should be considered for liver transplantation [5,6]. While mortality associated with WD varies, existing, limited, data suggest that the mortality rates among patients who are identified early and comply with treatment are comparable to those of the general population [7]. Unfortunately, diagnosis of WD is often delayed and there is frequently a lack of adherence to the necessary lifelong therapies among patients [8,9]. Even in patients with clinically stable and well-managed disease, WD is associated with disability and decreased quality of life [10]. A diagnosis of WD has also been shown to be associated with a significant increase in healthcare utilization and costs [11].

The National Inpatient Sample (NIS) is an all-payer inpatient database representative of hospital admissions in the US. In this retrospective cohort study, our primary outcomes were mortality, hospitalization costs, and length of stay (LOS) among patients with WD. The secondary outcomes were mortality, LOS, and hospitalization costs among WD patients following orthotopic liver transplantation (OLTx).

## Materials and methods

Data source

The Healthcare Cost and Utilization Project (HCUP) is a collection of databases, which includes the NIS database. NIS is the largest available dataset containing de-identified publicly available all-payer inpatient data derived from hospitalized patients in the US. The dataset is encoded by International Classification of Diseases (ICD) codes from over 1000 hospitals representing a stratified sample of approximately 20% of all hospital admissions in the US, excluding long-term acute care hospitals and rehabilitation hospitals. Primary and secondary diagnoses among the study population were identified using ICD, Ninth Revision, Clinical Modification (ICD-9-CM), and ICD, Tenth Revision, Clinical Modification (ICD-10-CM) codes. The Institutional Review Board (IRB) approval was not required as the HCUP-NIS database does not include patient identifiers.

Study design and patient population

This study was a retrospective analysis including the data of all patients in the database who were 18 years or older and hospitalized with a diagnosis of WD between 2007 and 2017. A primary diagnosis of WD was made if WD was listed as the first diagnosis code or admitting diagnosis. A secondary diagnosis of WD was established if WD was listed anywhere in the diagnosis coding (within 25 diagnoses) other than the initial code.

Definition of variables

Demographic variables we analyzed included age, gender, race/ethnicity, and patient’s insurance data. Outcome variables such as LOS, hospitalization costs, and in-hospital mortality were also obtained. A medical history comorbidity profile was created for each participant during the baseline period using ICD-9-CM/ICD-10-CM codes. The comorbidity profile included acute hepatitis, cirrhosis, liver failure, hemolytic anemia, cardiomyopathy, nephrocalcinosis, osteoarthritis, myocardial infarction, congestive heart failure (CHF), peripheral vascular disease (PVD), cerebrovascular accident (CVA)/transient ischemic attack (TIA), chronic obstructive pulmonary disease (COPD), connective tissue disease, peptic ulcer disease, diabetes mellitus (DM), chronic kidney disease (CKD), solid tumor, leukemia, lymphoma, and AIDS. Manifestations of liver disease (e.g., jaundice) and neurologic sequelae of WD (e.g., ataxia, dystonia, parkinsonism, tremor, mood disorders) were obtained using ICD coding. In addition, the total weighted Charlson Comorbidity Index (CCI) score was calculated for each patient.

Outcomes

The primary outcomes were mortality, LOS, and hospital costs in patients diagnosed with WD. Secondary outcomes included mortality, LOS, and hospital costs in WD patients following OLTx.

Statistical analysis

Bivariate analyses were performed using t-tests for continuous variables and Pearson’s chi-square tests for categorical variables, where two-sided p-values <0.05 were considered statistically significant. All analyses were performed using SAS version 9.4 (SAS Institute, Cary, NC).

## Results

Sample characteristics and comorbidity profiles

**Figure 1:**
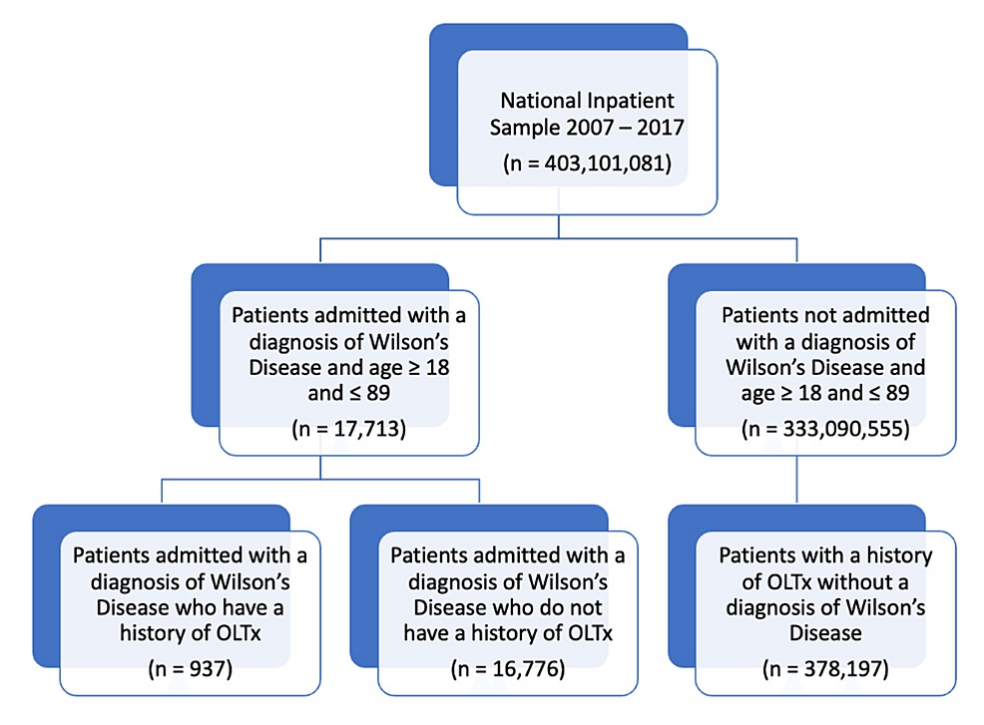
Diagram depicting study sample selection OLTx: orthotopic liver transplantation

A total of 17,713 weighted admissions with WD during the 10-year study period were analyzed (Figure 1). Overall, the mean age of the study population was 49.31 (SD: 39.22) years, with the highest proportion of patients belonging to the age group of 55-89 years (38.72%) (Table 1). Females represented 55.57% of the study population. Caucasian, black, and Hispanic patients accounted for 73.25%, 10.35%, and 9.65% of the study population, respectively. The primary healthcare payers were Medicare (37.88%) and private insurance (33.26%). Patients with WD had a higher expected prevalence of liver-related complications. Hemolytic anemia, connective tissue disease, and peptic ulcer disease were significantly more common among them. Kayser-Fleischer rings and other neuropsychiatric symptoms (e.g., ataxia, dystonia, parkinsonism) were also more common in this cohort.

**Table 1:** Baseline demographics of the study sample based on Wilson’s disease diagnosis A chi-square test was performed on all categorical variables. All continuous variables were tested for normality and resulted in non-parametric distributions *Wilcoxon rank-sum test performed SD: standard deviation; IQR: interquartile range

Study measure	Wilson's disease (yes) (n = 17,713)	Wilson's disease (no) - [total sample size (n = 333,090,555)]	P-value
Demographics			
Age, years			<0.0001*
Range	18 - 89	18 - 89	
Mean (SD)	49.31 (39.22)	55.98 (44.40)	
Median (IQR)	49 (35, 62)	58 (39, 72)	
Age group, years, n (%)			<0.0001
18 - 34	4,195 (23.68)	67,883,703 (20.38)	
35 - 44	3,154 (17.81)	33,955,902 (10.19)	
45 - 54	3,505 (19.79)	45,052,312 (13.53)	
55 - 89	6,858 (38.72)	186,198,638 (55.9)	
Sex, n (%)			<0.0001
Male	7,865 (44.43)	136,755,891 (41.09)	
Female	9,838 (55.57)	196,098,698 (58.91)	
Race/ethnicity, n (%)			<0.0001
White	11,771 (73.25)	202,299,033 (67.81)	
Black	1,663 (10.35)	44,904,162 (15.05)	
Hispanic	1,550 (9.65)	32,416,567 (10.87)	
Asian or Pacific Islander	413 (2.57)	7,466,118 (2.5)	
Native American	70 (0.43)	1,996,112 (0.67)	
Other	602 (3.75)	9,234,328 (3.1)	
Primary payer, n (%)			<0.0001
Medicare	6,688 (37.88)	146,255,549 (44)	
Medicaid	3,510 (19.88)	55,937,113 (16.83)	
Private insurance	5,874 (33.26)	100,519,236 (30.24)	
Self-pay	845 (4.79)	16,934,770 (5.09)	
No charge	121 (0.68)	1,747,795 (0.53)	
Other	621 (3.52)	11,015,433 (3.31)	

Comparison of outcomes between WD and non-WD patients

Outcomes, including mortality, LOS, and inpatient hospitalization costs, are summarized in Table 2. The WD cohort had a significantly longer LOS (7.34 vs. 4.62 days; p<0.0001), a higher mortality rate (2.51% vs. 2.05%; p<0.0001), and higher mean hospitalization costs ($61,648.45 vs. $33,608.94; p<0.0001).

**Table 2:** Prevalence of comorbidities and outcomes, including mortality, length of stay, and hospitalization costs, in patients with and without a diagnosis of Wilson’s disease A chi-square test was performed on all categorical variables. All continuous variables were tested for normality and resulted in non-parametric distributions *Fisher's exact test performed; **Wilcoxon rank-sum test performed The prevalence of the following comorbidities did not meet statistical significance (p>0.05): depression, mood disorders, nephrocalcinosis The prevalence of the following comorbidities was significantly (p<0.05) higher among the non-WD cohort: anxiety, cardiomyopathy, osteoarthritis, myocardial infarction, CHF (PND/DOE), peripheral vascular disease, CVA/TIA, dementia, COPD, uncomplicated diabetes mellitus, diabetes mellitus with end-organ damage, hemiplegia, moderate to severe CKD, localized solid tumor, solid tumor metastasis, leukemia, lymphoma SD: standard deviation; IQR: interquartile range; CHF (PND/DOE): congestive heart failure (paroxysmal nocturnal dyspnea/dyspnea on exertion); CVA/TIA: cerebrovascular accident/transient ischemic attack; COPD: chronic obstructive pulmonary disease; CKD: chronic kidney disease; AIDS: acquired immunodeficiency syndrome; CCI: Charlson Comorbidity Index

Comorbidities			
	Wilson's disease (yes) (n = 17,713)	Wilson's disease (no) - [total sample size (n = 333,090,555)]	P-value
Acute hepatitis, n (%)			<0.0001
No	17,534 (98.99)	332,683,022 (99.88)	
Yes	179 (1.01)	407,533 (0.12)	
Cirrhosis, n (%)			<0.0001
No	15,146 (85.51)	329,847,482 (99.03)	
Yes	2,567 (14.49)	3,243,073 (0.97)	
Liver failure, n (%)			<0.0001
No	16,822 (94.97)	331,865,849 (99.63)	
Yes	892 (5.03)	1,224,706 (0.37)	
Jaundice, n (%)			<0.0001
No	17,381 (98.12)	332,227,050 (99.74)	
Yes	333 (1.88)	863,506 (0.26)	
Hemolytic anemia, n (%)			<0.0001
No	17,532 (98.98)	332,945,196 (99.96)	
Yes	181 (1.02)	145,360 (0.04)	
Portal hypertension, n (%)			<0.0001
No	16,055 (90.64)	331,145,852 (99.42)	
Yes	1,658 (9.36)	1,944,703 (0.58)	
Ataxia, n (%)			<0.0001
No	17,677 (99.8)	333,056,806 (99.99)	
Yes	36 (0.2)	33,749 (0.01)	
Dystonia, n (%)			<0.0001
No	17,648 (99.63)	333,053,640 (99.99)	
Yes	65 (0.37)	36,915 (0.01)	
Secondary parkinsonism, n (%)			<0.0001
No	17,703 (99.94)	333,049,899 (99.99)	
Yes	10 (0.06)	40,656 (0.01)	
Tremor, n (%)			<0.0001
No	17,608 (99.41)	332,319,590 (99.77)	
Yes	105 (0.59)	77,0965 (0.23)	
Anxiety, n (%)			0.03
No	17,378 (98.11)	326,015,743 (97.88)	
Yes	335 (1.89)	7,074,812 (2.12)	
Behavioral disorders, n (%)			1*
No	17,713 (100)	333,090,495 (100)	
Yes	0 (0)	60 (0)	
Depression, n (%)			0.24
No	17,318 (97.77)	325,219,142 (97.64)	
Yes	395 (2.23)	7,871,413 (2.36)	
Impulse disorders, n (%)			1*
No	17,713 (100)	333,051,515 (99.99)	
Yes	0 (0)	39,040 (0.01)	
Mood disorders, n (%)			0.98
No	17,698 (99.92)	332,806,705 (99.91)	
Yes	15 (0.08)	28,3850 (0.09)	
Cardiomyopathy, n (%)			<0.0001
No	17,693 (99.89)	331,721,565 (99.59)	
Yes	20 (0.11)	1,368,990 (0.41)	
Hypercalciuria, n (%)			-
No	17,713 (100)	333,090,555 (100)	
Yes	0 (0)	0 (0)	
Kayser-Fleischer rings, n (%)			<0.0001
No	17,529 (98.96)	333,090,545 (100)	
Yes	184 (1.04)	10 (0)	
Nephrocalcinosis, n (%)			0.1
No	17,698 (99.92)	332,905,963 (99.94)	
Yes	15 (0.08)	184,592 (0.06)	
Osteoarthritis, n (%)			<0.0001
No	17,593 (99.32)	329,171,532 (98.82)	
Yes	120 (0.68)	3,919,023 (1.18)	
Myocardial infarction, n (%)			<0.0001
No	17,204 (97.12)	308,639,476 (92.66)	
Yes	510 (2.88)	24,451,079 (7.34)	
CHF (PND/DOE), n (%)			<0.0001
No	16,375 (92.44)	289,094,116 (86.79)	
Yes	1,338 (7.56)	43,996,439 (13.21)	
Peripheral vascular disease, n (%)			<0.0001
No	17,599 (99.36)	327,994,788 (98.47)	
Yes	114 (0.64)	5,095,768 (1.53)	
CVA/TIA, n (%)			<0.0001
No	16,856 (95.16)	311,890,299 (93.64)	
Yes	857 (4.84)	21,200,257 (6.36)	
Dementia, n (%)			<0.0001
No	17,462 (98.58)	323,793,131 (97.21)	
Yes	251 (1.42)	9,297,424 (2.79)	
COPD, n (%)			<0.0001
No	16,963 (95.77)	308,506,673 (92.62)	
Yes	750 (4.23)	24,583,882 (7.38)	
Connective tissue disease, n (%)			<0.0001
No	16,978 (95.85)	322,856,664 (96.93)	
Yes	735 (4.15)	10,233,891 (3.07)	
Peptic ulcer disease, n (%)			<0.0001
No	17,281 (97.56)	329,251,658 (98.85)	
Yes	433 (2.44)	3,838,897 (1.15)	
Mild liver disease, n (%)			<0.0001
No	16,459 (92.92)	329,104,466 (98.8)	
Yes	1,255 (7.08)	3,986,089 (1.2)	
Moderate to severe liver disease, n (%)			<0.0001
No	15,721 (88.76)	330,693,312 (99.28)	
Yes	1,992 (11.24)	2,397,244 (0.72)	
Uncomplicated diabetes mellitus, n (%)			<0.0001
No	15,570 (87.9)	281,226,238 (84.43)	
Yes	2,143 (12.1)	51,864,317 (15.57)	
Diabetes mellitus with end-organ damage, n (%)			<0.0001
No	16,951 (95.7)	314,277,674 (94.35)	
Yes	762 (4.3)	18,812,881 (5.65)	
Hemiplegia, n (%)			<0.0001
No	17,637 (99.57)	330,223,784 (99.14)	
Yes	76 (0.43)	2,866,771 (0.86)	
Moderate to severe CKD, n (%)			<0.0001
No	16,422 (92.71)	304,318,770 (91.36)	
Yes	1,291 (7.29)	28,771,785 (8.64)	
Localized solid tumor, n (%)			<0.0001
No	16,092 (90.85)	290,863,364 (87.32)	
Yes	1,621 (9.15)	42,227,191 (12.68)	
Solid tumor with metastasis, n (%)			<0.0001
No	17,322 (97.79)	322,684,230 (96.88)	
Yes	391 (2.21)	10,406,325 (3.12)	
Leukemia, n (%)			0.003
No	17,571 (99.2)	329,666,709 (98.97)	
Yes	142 (0.8)	3,423,846 (1.03)	
Lymphoma, n (%)			<0.0001
No	17,650 (99.65)	331,137,260 (99.41)	
Yes	63 (0.35)	1,953,295 (0.59)	
AIDS, n (%)			<0.0001
No	17,572 (99.2)	331,593,859 (99.55)	
Yes	141 (0.8)	1,496,697 (0.45)	
CCI total score			<0.0001**
Range	0 - 17	0 - 22	
Mean (SD)	2.35 (5.51)	2.91 (6.09)	
Median (IQR)	2 (0, 4)	3 (0, 5)	
Primary outcomes			
Mortality, n (%)			<0.0001
No	17,260 (97.49)	326,066,094 (97.95)	
Yes	444 (2.51)	6,814,854 (2.05)	
Length of stay (days)			<0.0001**
Range	0 - 298	0 - 365	
Mean (SD)	7.34 (23.95)	4.72 (14.28)	
Median (IQR)	4 (2, 8)	3 (2, 5)	
Total cost (US Dollars)			<0.0001**
Range	$625 - $2,599,217	$100 - $9,999,999	
Mean (SD)	$61,648.45 ($268,782.79)	$40,666.32 ($151,365.74)	
Median (IQR)	$29,433 ($14,821, $61,181)	$22,728 ($11,994, $45,000)	

Comparison of outcomes among WD patients following OLTx

Of the 17,713 patients in the WD cohort, 937 patients had undergone OLTx (Table 3). Hispanic ethnicity (15.17% vs. 9.34%) and utilization of private insurance (40.23% vs. 32.87%) were more common among the WD patients who had undergone OLTx. WD patients who had undergone OLTx had a higher prevalence of anxiety, depression, osteoarthritis, and jaundice. Connective tissue disorder was more common among the WD patients (with and without a history of OLTx). WD patients who had not undergone OLTx had a higher rate of acute hepatitis, liver failure, liver disease (mild and moderate to severe), portal hypertension, cirrhosis, peptic ulcer disease, ataxia, dystonia, tremor, and evidence of Kayser-Fleisher rings. WD patients who had undergone OLTx had a lower degree of comorbidity burden compared to the WD patients without a history of OLTx and non-WD patients who had undergone OLTx (mean CCI: 1.63 vs. 2.39 vs. 3.72; p<0.0001).

**Table 3:** Baseline demographics of Wilson’s disease patients by liver transplant status A chi-square test was performed on all categorial variables unless otherwise noted. All continuous variables were tested for normality and resulted in non-parametric distributions ^†^P-value describing the observed difference between the WD + OLTx cohort vs. the WD – OLTx cohort. ^††^P-value describing the observed difference between the WD + OLTx vs. WD – OLTx vs. Non-WD + OLTx cohorts. *Wilcoxon rank-sum test performed. **Fisher's exact test performed. ***Kruskal-Wallis test performed SD: standard deviation; IQR: interquartile range; OLTx: orthotopic liver transplantation; WD: Wilson's disease

Study measure	Wilson's disease = yes, OLTx = yes, n = 937	Wilson's disease = yes, OLTx = no, n = 16,776	Wilson's disease = no, OLTx = yes, n = 378,197	^†^P-value: WD (Y)/OLTx (Y) vs. WD (Y)/OLTx (N)	^††^P-value: WD (Y)/OLTx (Y) vs. WD (Y)/OLTx (N) vs. WD (N)/OLTx (Y)
Demographics					
Age, years				<0.0001*	<0.0001***
Range	18 - 68	18 - 89	18 - 89		
Mean (SD)	40.16 (30.51)	49.82 (39.34)	57.62 (29.14)		
Median (IQR)	40 (28, 52)	50 (36, 62)	60 (52, 66)		
Age group, years, n (%)				<0.0001	<0.0001
18 - 34	324 (34.52)	3,872 (23.08)	29,399 (7.77)		
35 - 44	241 (25.66)	2,914 (17.37)	21,756 (5.75)		
45 - 54	190 (20.32)	3,315 (19.76)	69,059 (18.26)		
55 - 89	183 (19.5)	6,676 (39.79)	257,984 (68.21)		
Year, n (%)				<0.0001	<0.0001
2007	78 (8.28)	1,058 (6.31)	24,973 (6.6)		
2008	80 (8.49)	1,368 (8.16)	33,247 (8.79)		
2009	127 (13.54)	1,553 (9.25)	32,442 (8.58)		
2010	81 (8.66)	1,715 (10.22)	33,639 (8.89)		
2011	67 (7.16)	1,727 (10.29)	35,771 (9.46)		
2012	70 (7.47)	1,905 (11.36)	32,290 (8.54)		
2013	85 (9.07)	1,700 (10.13)	33,625 (8.89)		
2014	70 (7.47)	1,975 (11.77)	36,120 (9.55)		
2015	70 (7.47)	1,720 (10.25)	36,565 (9.67)		
2016	100 (10.67)	985 (5.87)	38,405 (10.15)		
2017	110 (11.74)	1,070 (6.38)	41,120 (10.87)		
Sex, n (%)				0.36	<0.0001
Male	403 (42.99)	7,462 (44.51)	227,494 (60.18)		
Female	534 (57.01)	9,303 (55.49)	150,549 (39.82)		
Race/ethnicity, n (%)				0.01**	<0.0001
White	578 (67.63)	11,193 (73.57)	246,029 (71.74)		
Black	64 (7.49)	1,599 (10.51)	31,258 (9.11)		
Hispanic	130 (15.17)	1,421 (9.34)	43,730 (12.75)		
Asian or Pacific Islander	15 (1.76)	398 (2.61)	9,466 (2.76)		
Native American	0 (0)	70 (0.46)	1,943 (0.57)		
Other	68 (7.95)	534 (3.51)	10,507 (3.06)		
Primary payer, n (%)				0.03**	<0.0001
Medicare	318 (34.07)	6,371 (38.09)	208,607 (55.27)		
Medicaid	209 (22.45)	3,301 (19.73)	40,392 (10.7)		
Private insurance	375 (40.23)	5,499 (32.87)	116,683 (30.91)		
Self-pay	20 (2.18)	825 (4.93)	3,717 (0.98)		
No charge	0 (0)	121 (0.72)	294 (0.08)		
Other	10 (1.07)	611 (3.65)	7,762 (2.06)		

Differences in outcomes, including mortality, LOS, and inpatient hospitalization costs, are presented in Table 4. The mortality rate was lower in the WD + OLTx cohort (1.07% vs. 2.59% vs. 1.91%; p<0.0001). The WD + OLTx cohort was associated with a significantly shorter LOS (mean LOS: 5.64 vs. 7.44 days; p<0.0001) compared to the WD - OLTx cohort. Hospital costs were lower in the WD + OLTx cohort compared to the WD - OLTx cohort; however, this did not reach statistical significance (mean hospitalization cost: $53,053.99 vs. $62,132.13; p=0.37). The WD + OLTx cohort and non-WD + OLTx cohorts had similar mean LOS and hospitalization costs.

**Table 4:** Prevalence of comorbidities and outcomes, including mortality, length of stay, and hospitalization costs, among Wilson’s disease patients with a history of liver transplant, Wilson’s disease patients without a history of liver transplant, and patients with a history of liver transplant without a diagnosis of Wilson’s disease A chi-square test was performed on all categorial variables unless otherwise noted. All continuous variables were tested for normality and resulted in non-parametric distributions ^†^P-value describing the observed difference between the WD + OLTx cohort vs. the WD – OLTx cohort. ^††^P-value describing the observed difference between the WD + OLTx vs. WD – OLTx vs. Non-WD + OLTx cohorts. *Fisher's exact test performed. **Wilcoxon rank-sum test performed. ***Kruskal-Wallis test performed The prevalence of the following comorbidities did not reach statistical significance (p>0.05): Parkinson’s disease, impulse disorders, mood disorders, nephrocalcinosis, hemiplegia, leukemia The prevalence of the following comorbidities was significantly higher (p<0.05) among the WD + OLTx cohort: anxiety, depression, osteoarthritis, peripheral vascular disease, CVA/TIA, lymphoma The prevalence of the following comorbidities was significantly higher (p<0.05) among the non-WD + OLTx cohort: cardiomyopathy, myocardial infarction, CHF (PND/DOE), COPD, localized solid tumor, and solid tumor with metastasis SD: standard deviation; IQR: interquartile range; CHF (PND/DOE): congestive heart failure (paroxysmal nocturnal dyspnea/dyspnea on exertion); CVA/TIA: cerebrovascular accident/transient ischemic attack; COPD: chronic obstructive pulmonary disease; CKD: chronic kidney disease; AIDS: acquired immunodeficiency syndrome; CCI: Charlson Comorbidity Index; OLTx: orthotopic liver transplantation; WD: Wilson's disease

Study measure	Wilson's disease = yes, OLTx = yes, n = 937	Wilson's disease = yes, OLTx = no, n = 16,776	Wilson's disease = no, OLTx = yes, n = 378,197	^†^P-value: WD (Y)/OLTx (Y) vs. WD (Y)/OLTx (N)	^††^P-value: WD (Y)/OLTx (Y) vs. WD (Y)/OLTx (N) vs. WD (N)/OLTx (Y)
Comorbidities					
Acute hepatitis, n (%)	5 (0.55)	174 (1.04)	730 (0.19)	0.15	<0.0001
Cirrhosis, n (%)	65 (6.88)	2,502 (14.92)	23,019 (6.09)	<0.0001	<0.0001
Liver failure, n (%)	5 (0.53)	887 (5.29)	4,113 (1.09)	<0.0001	<0.0001
Jaundice, n (%)	25 (2.65)	308 (1.83)	3,661 (0.97)	0.07	<0.0001
Hemolytic anemia, n (%)	5 (0.54)	176 (1.05)	543 (0.14)	0.13	<0.0001
Portal hypertension, n (%)	15 (1.6)	1,643 (9.8)	11,654 (3.08)	<0.0001	<0.0001
Ataxia, n (%)	0 (0)	36 (0.22)	36 (0.01)	1*	<0.0001
Dystonia, n (%)	0 (0)	65 (0.39)	55 (0.01)	1*	<0.0001
Secondary parkinsonism, n (%)	0 (0)	10 (0.06)	50 (0.01)	1*	0.1
Tremor, n (%)	6 (0.6)	100 (0.59)	1,433 (0.38)	0.98	<0.0001
Anxiety, n (%)	30 (3.2)	305 (1.82)	8,850 (2.34)	0.003	<0.0001
Behavioral disorders, n (%)	0 (0)	0 (0)	0 (0)	-	-
Depression, n (%)	35 (3.73)	360 (2.15)	13,469 (3.56)	0.001	<0.0001
Impulse disorders, n (%)	0 (0)	0 (0)	5 (0)		0.98
Mood disorders, n (%)	0 (0)	15 (0.09)	295 (0.08)	1*	0.91
Cardiomyopathy, n (%)	0 (0)	20 (0.12)	1,530 (0.4)	1*	0.02
Hypercalciuria, n (%)	0 (0)	0 (0)	0 (0)	-	-
Kayser-Fleischer rings, n (%)	0 (0)	184 (1.10)	0 (0)	0.26*	<0.0001
Nephrocalcinosis, n (%)	0 (0)	15 (0.09)	565 (0.15)	1*	0.58
Osteoarthritis, n (%)	20 (2.13)	100 (0.6)	4,245 (1.12)	<0.0001	<0.0001
Myocardial infarction, n (%)	20 (2.13)	490 (2.92)	20,778 (5.49)	0.16	<0.0001
CHF (PND/DOE), n (%)	65 (6.91)	1,274 (7.59)	44,497 (11.77)	0.44	<0.0001
Peripheral vascular disease, n (%)	0 (0)	114 (0.68)	3,192 (0.84)	0.63*	0.26
CVA/TIA, n (%)	34 (3.65)	823 (4.91)	17,875 (4.73)	0.08	0.16
Dementia, n (%)	0 (0)	251 (1.5)	2,960 (0.78)	0.11*	<0.0001
COPD, n (%)	25 (2.67)	725 (4.32)	16,439 (4.35)	0.01	0.04
Connective tissue disease, n (%)	40 (4.27)	695 (4.14)	9,397 (2.48)	0.85	<0.0001
Peptic ulcer disease, n (%)	20 (2.13)	413 (2.46)	5,026 (1.33)	0.53	<0.0001
Mild liver disease, n (%)	20 (2.09)	1,235 (7.36)	23,434 (6.2)	<0.0001	<0.0001
Moderate to severe liver disease, n (%)	24 (2.56)	1,968 (11.73)	16,407 (4.34)	<0.0001	<0.0001
Uncomplicated diabetes mellitus, n (%)	98 (10.46)	2,045 (12.19)	105,105 (27.79)	0.11	<0.0001
Diabetes mellitus with end-organ damage, n (%)	78 (8.37)	684 (4.08)	43,845 (11.59)	<0.0001	<0.0001
Hemiplegia, n (%)	0 (0)	76 (0.45)	1,862 (0.49)	1*	0.58
Moderate to severe CKD, n (%)	214 (22.8)	1,077 (6.42)	135,965 (35.95)	<0.0001	<0.0001
Localized solid tumor, n (%)	93 (9.92)	1,528 (9.11)	67,302 (17.8)	0.4	<0.0001
Solid tumor with metastasis, n (%)	5 (0.53)	386 (2.3)	9,612 (2.54)	0.0003	<0.0001
Leukemia, n (%)	0 (0)	142 (0.85)	2,385 (0.63)	0.4*	0.14
Lymphoma, n (%)	15 (1.55)	48 (0.29)	3,302 (0.87)	<0.0001	<0.0001
AIDS, n (%)	0 (0)	141 (0.84)	739 (0.2)	0.4*	<0.0001
CCI total score				<0.0001**	<0.0001***
Range	0 - 9	0 - 17	0 - 19		
Mean (SD)	1.63 (4.97)	2.39 (5.53)	3.72 (5.62)		
Median (IQR)	1 (0, 2)	2 (0, 4)	3 (2, 5)		
Primary outcomes					
Mortality, n (%)	10 (1.07)	434 (2.59)	7,231 (1.91)	0.004	<0.0001
Length of stay, days				<0.0001**	<0.0001***
Range	0 - 175	0 - 298	0 - 295		
Mean (SD)	5.64 (30.2)	7.44 (23.54)	5.63 (17.41)		
Median (IQR)	3 (2, 5)	5 (3, 8)	4 (2, 6)		
Total cost (UD Dollars)				0.37**	0.56***
Range	$2,658 - $1,348,927	$625 - $2,599,217	$199 - $4,239,568		
Mean (SD)	$53,053.99 ($249,312.32)	$62,132.13 ($269,831.72)	$53,290.24 ($217,248.53)		
Median (IQR)	$27,354 ($14,361, $56,810)	$29,508 ($14,869, $61,406)	$29,039 ($15,526, $56,630)		

## Discussion

In this retrospective database analysis, we evaluated clinical characteristics, outcomes, and inpatient costs associated with 17,713 patients with WD compared to 333,090,555 patients without WD. We further stratified the WD population into 937 patients who had undergone OLTx and 16,776 who had not.

Our results showed significantly worse inpatient mortality in WD patients (2.51%) compared to the non-WD cohort. The estimated mortality rate of patients with WD widely varies in the literature globally, ranging from 1.8% to 21.1% [12-19]. Mortality associated with WD has been shown to be higher compared to healthy controls [13,15]; however, the mortality rate among treatment-compliant WD patients may be comparable to that of the general population [12,7,20]. Since we utilized the NIS database, our mortality rate was inclusive of all WD patients regardless of disease stage, compliance, and response to treatment, which explains the more heterogeneous representation of disease-related mortality differences in our findings.

WD was also associated with a significantly increased LOS and higher costs of hospitalization (mean: $61,648.45 vs. $40,666.32; p<0.0001). Liver disease and cirrhosis were more common in the WD cohort, which may contribute to the higher healthcare utilization. LOS and inpatient cost for patients with chronic liver disease (CLD) is high due to several factors including the likelihood of needing advanced procedures and escalation of care to ICUs [21,22]. Interestingly, a previous study using the US commercial claims database has described higher healthcare costs among WD patients compared to patients with other CLDs [11]. There are likely disease-specific complications that inflate inpatient costs in WD patients particularly. Further studies are required to understand these factors better to better mitigate the cost burden. 

In our cohort, when comparing WD + OLTx patients to WD - OLTx patients, those who had undergone OLTx had a 58.68% reduction in inpatient mortality compared to WD patients who had not undergone OLTx. Other studies have also shown OLTx to improve mortality rates in patients with WD [23-25]. Liver transplantation is the only effective and functional cure for WD and is generally considered in patients with end-stage liver disease and/or fulminant hepatic failure who no longer respond to medical therapy [23,26]. Interestingly, our cohort of patients with WD who had undergone OLTx also had decreased LOS (mean LOS: 5.64 vs. 7.44 days; p<0.0001) and inpatient cost (mean hospitalization cost: $53,053.99 vs. $62,132.13; p=0.37) as compared to WD patients without OLTx. Despite the improved mortality rates and financial benefits of transplants, WD patients represent a very small minority of patients who undergo liver transplants, with epidemiologic data suggesting that only 1.5% of transplants in adults are due to WD [21]. Our data may suggest that early transplant consideration in patients with WD is beneficial for both the patient and the healthcare payer.

This study has some limitations, most of which are associated with the use of the NIS database. Inaccuracies in capturing ICD-9 and ICD-10 coding can be considered the primary source of limitation. NIS also does not provide longitudinal outcomes data following discharge; hence, although we reported mortality rates in our study population, we were only able to report inpatient mortality rather than the true mortality rate. Due to the nature of the database, primary causes for admission and mortality were unable to be extracted. Additionally, we were unable to characterize the reasons for the duration of stay or hospitalization cost, which may be unrelated to clinical needs and rather driven by disposition or social issues. Despite these limitations, the use of the NIS dataset provides multiple aspects of strength since it is the largest publicly available all-payer inpatient care database and is representative of all regions throughout the US. Through this extensive dataset, we were able to access a significantly larger sample of patients diagnosed with WD to perform analyses and generate meaningful data not otherwise available in the literature (e.g., meta-analyses, systematic reviews, or smaller cohort analyses).

## Conclusions

Our study demonstrates that a diagnosis of WD is associated with higher inpatient mortality, LOS, and hospitalization costs compared to patients without this diagnosis. Liver transplantation offers a promising solution as it dramatically improves survival rates, shortens LOS, and reduces inpatient costs. Diagnosing and treating WD is a challenging task due to its rarity, delayed diagnosis, and comorbid neuropsychiatric symptoms that can hinder adherence to current treatment regimens. Our findings highlight the importance of effective interventions to prevent potential complications and reduce the burden of WD on patient outcomes, healthcare utilization, and costs. Further studies are warranted to improve upon our current guidelines and systems to identify WD patients early and guide them toward appropriate treatment.
